# Proceedings of the 5th Annual United States Army Institute of Surgical Research Summer Undergraduate Research Internship Program 2017

**DOI:** 10.1186/s12967-017-1316-3

**Published:** 2017-11-27

**Authors:** Ryan Leone, Katie J. Jensen, Claire Abijay, Troy Dolmetsch, Natalie Koons, Daniel N. Darlington, Andrew P. Cap, Xiaowu Wu, Christopher P. Delavan, Maryanne C. Herzig, Barbara A. Christy, Kelley M. Kempski, August N. Blackburn, Robert A. De Lorenzo, Megan B. Blackburn, Matthew C. Donald, Harold G. Klemcke, Brenna K. Harrington, Celestine J. He, Belinda I. Gómez, Tony Chao, Joshua S. Little, Tiffany C. Heard, Michael A. Dubick, David M. Burmeister, Amy Xu, Kerfoot Walker III, Arezoo Mohammadipoor, Luis Rodriguez, Teryn Roberts, Andriy Batchinsky, Leopoldo Cancio, Ben Antebi, Ryan A. Walford, Colby S. McIntosh, Grantham C. Peltier, Umang Sharma, Robbie K. Montgomery, Michael A. Meledeo, James A. Bynum, Sarah Lovelace, Larry Estlack, Katherine Jensen, Lexi Kazen, Lee C. Mangum, Gerardo R. Garcia, Kevin S. Akers

**Affiliations:** 1 Univeristy of Pennsylvania, Philadelphia, PA 19104 USA; 2 Rice University, Houston, TX 77005 USA; 3 Georgetown University, Washington, DC 20057 USA; 4 East Tennessee State University, Johnson City, TN 37614 USA; 5 Mercyhurst University, Erie, PA 16504 USA; 6 US Army Institute of Surgical Research, JBSA Fort Sam Houston, TX 78234 USA; 7 Tactical Combat Casualty Care Research Task Area, US Army Institute of Surgical Research, JBSA Fort Sam Houston, TX 78234 USA; 8 Department of Biomedical Engineering, University of Delaware, Newark, DE 19716 USA; 9 South Texas Diabetes and Obesity Institute, University of Rio Grande Valley, Brownsville, TX 78520 USA; 10 University of Texas Health Science Center at San Antonio, San Antonio, TX 78229 USA; 11 Mississippi State University, Mississippi State, MS 39762 USA; 12 Damage Control Resuscitation Task Area, US Army Institute of Surgical Research, JBSA Fort Sam Houston, TX 78234 USA; 13 Stanford University, Stanford, CA USA; 14 Oak Ridge Institute for Science and Education, Oak Ridge, TN USA; 15 The Geneva Foundation, Tacoma, WA USA; 16 Blood and Coagulation Task Area, US Army Institute of Surgical Research, JBSA Fort Sam Houston, TX 78234 USA; 17 Dwight Look College of Engineering, Department of Biomedical Engineering, Texas A&M University, College Station, TX 77840 USA; 18 Trinity University, San Antonio, TX 78212 USA; 19 University of Wisconsin, Madison, WI USA; 20 Brooke Army Medical Center, JBSA Fort Sam Houston, TX USA; 21 Department of Biological Sciences, Northwestern University, Evanston, IL 60201 USA

## I1 Proceedings of the 5th Annual United States Army Institute of Surgical Research Summer Undergraduate Research Internship Program 2017

### Ryan Leon^1^, Katie Jensen^2^, Claire Abijay^3^, Troy Dolmetsch^4^, Natalie Koons^5^, Daniel N. Darlington^6^, Andrew Cap^6^, Xiaowu Wu^6^, Christopher P. Delavan^6^, Maryanne C. Herzig^6^, Barbara A. Christy^6^, Kelley M. Kempski^7,8^, August N. Blackburn^9^, Robert A. De Lorenzo ^7,10^, Megan B. Blackburn^7^, Matthew C. Donald^11^, Harold G. Klemcke^7^, Brenna K. Harrington^12^, Celestine J. He^12,21^, Belinda I. Gómez^12^, Tony Chao^12^, Joshua S. Little^12^, Tiffany C. Heard^12^, Michael A. Dubick^12^, David M. Burmeister^12^, Amy Xu^6,13^, Kerfoot Walker III ^6,14^, Arezoo Mohammadipoor^6,14^, Luis Rodriguez ^6,14^, Teryn Roberts ^6,15^, Andriy Batchinsky ^6,15^, Leopoldo Cancio ^6^, Ben Antebi ^6^, Ryan A. Walford^16,17^, Colby S. McIntosh^16^, Grantham C. Peltier^16^, Umang Sharma^16^, Robbie K. Montgomery^16^, Michael A. Meledeo^16^, James A. Bynum^16^, Sarah Lovelace^18^, Larry Estlack^6^, Lexi Kazen^19^, Lee C. Mangum^6^, Gerardo R. Garcia^6^, Kevin S. Akers^6,20^, Whitney Greene^6^, Lauren Cornell^6^

#### ^1^Univeristy of Pennsylvania, Philadelphia, PA 19104, USA; ^2^Rice University, Houston, TX 77005, USA; ^3^Georgetown University, Washington, DC 20057, USA; ^4^East Tennessee State University, Johnson City, TN 37614, USA; ^5^Mercyhurst University, Erie, PA 16504, USA; ^6^US Army Institute of Surgical Research, JBSA Fort Sam Houston, TX 78234, USA; ^7^Tactical Combat Casualty Care Research Task Area, US Army Institute of Surgical Research, JBSA Fort Sam Houston, TX 78234, USA; ^8^Department of Biomedical Engineering, University of Delaware, Newark, DE 19716, USA; ^9^South Texas Diabetes and Obesity Institute, University of Rio Grande Valley, Brownsville, TX 78520, USA; ^10^University of Texas Health Science Center at San Antonio, San Antonio, TX 78229, USA; ^11^Mississippi State University, Mississippi State, MS 39762, USA; ^12^Damage Control Resuscitation Task Area, US Army Institute of Surgical Research, JBSA Fort Sam Houston, TX 78234, USA; ^13^Stanford University, Stanford, CA, USA; ^14^Oak Ridge Institute for Science and Education, Oak Ridge, TN, USA; ^15^The Geneva Foundation, Tacoma, WA, USA; ^16^Blood and Coagulation Task Area, US Army Institute of Surgical Research, JBSA Fort Sam Houston, TX 78234, USA; ^17^Dwight Look College of Engineering, Department of Biomedical Engineering, Texas A&M University, College Station, TX 77840, USA; ^18^Trinity University, San Antonio, TX 78212, USA; ^19^University of Wisconsin, Madison, WI, USA; ^20^Brooke Army Medical Center, JBSA Fort Sam Houston, TX, USA; ^21^Department of Biological Sciences, Northwestern University, Evanston, IL 60201, USA

##### **Correspondence:** Lauren Cornell (lauren.e.cornell.ctr@mail.mil)

*Journal of Translational Medicine* 2017, **15(Supp 4)**:I1

**Description of the Summer Internship Program**

Since its establishment in 1943, the US Army Institute of Surgical Research (USAISR) has facilitated the development of innovative technologies that improve combat casualty care. The Summer Undergraduate Internship Program was created to expose students interested in biomedical research to the medical challenges that are addressed by scientific investigators affiliated with the Department of Defense (DoD).

After being selected from a pool of nearly 1500 applicants, the 13 participants in the 2017 program were paired with experienced research mentors in one of the following task areas: Resuscitation, Hemorrhage Control, Burn Injury, Blood and Coagulation, Ocular Trauma, Dental Trauma, Pre-hospital Trauma Care, Intensive Care, Pain, Regenerative Medicine, and Extremity Trauma.

Beyond their lab experiences, the interns shadowed physicians during rounds in the USAISR Burn Center Intensive Care Unit, toured the Center for the Intrepid, gardened at the Warrior and Family Support Center, participated in a weekly journal club, and learned about careers in research, medicine, and military service. This year’s 10-week program took place from May 24th to August 2nd.

**Eligibility**

Successful applicants to the USAISR Internship Program must have completed their first year of undergraduate study in an accredited Bachelor’s degree program. Applicants pursuing STEM majors are preferred. All applicants must be current US Citizens to qualify for the program.

**Meeting format**

After 10 weeks of research in their respective departments, participants compiled their research findings and participated in a poster session at the USAISR presenting to the USAISR, Naval Medical Research Unit-San Antonio, and the San Antonio Military Medical Center scientific community. Attendees include clinicians, military personnel, research fellows, and principal investigators (PIs).

**Awards**

Interns who successfully completed the program were recognized with Certificates of Appreciation by the USAISR’s Director of Research, Dr. Anthony Pusateri. Research conducted by the students contributed to the advancement of healthcare for the soldiers who are wounded in combat.

**Conclusions**

The USAISR Summer Internship Program provided a unique opportunity for students to access the specialized resources of a military research facility, receive mentorship from accomplished investigators, learn about scientific investigation first-hand, and understand the complex nature of the combat injuries sustained by the men and women in our military. As the students conducted research and took training courses, they acquired a thorough understanding of safety protocols, laboratory skills, and the underlying biological mechanisms that control bodily responses to traumatic injuries. They also learned how to critically read scientific articles, compose lab notes, and present papers at the weekly journal club meetings. These lessons were taught through close instruction and supervision from mentors as the students witnessed the integration of technology, medicine, and basic science that will lead to improved care for the combat-wounded.

**Declarations**

**Acknowledgements**

We would like to express our gratitude to the USAISR Research Directorate for their sponsorship of this internship program and publication. Specifically we would like to thank, Director of Research Dr. Anthony Pusateri, Deputy Director of Research Capt. Melissa Kottke, and Ms. Susan Walker. We would also like to acknowledge the summer intern program coordinator, Dr. David Burmeister, student coordinator Sherwin Cruz, and the individual departments and PIs who committed time to mentoring students. The financial support for this program is due to a contract between the USAISR and the Oak Ridge Institute for Science and Education. Institutional Editors: Lauren Cornell, M.S and Whitney Greene, Ph.D.

**Disclaimers**

The opinions and assertions contained herein are the private views of the authors and are not to be construed as official or reflecting the views of the Department of Defense or Departments of the Army. This research debuted below was funded by the US Army Medical Research and Materiel Command. These studies were conducted under protocols reviewed and approved by the US Army Medical Research and Materiel Command Institutional Review Board and in accordance with the approved protocols.

**Funding**

Publication charges for this supplement were funded by the USAISR.

**Consent**

The authors have written informed consent from the people in Figure 1 to publish the image.

**Fig. 1:**
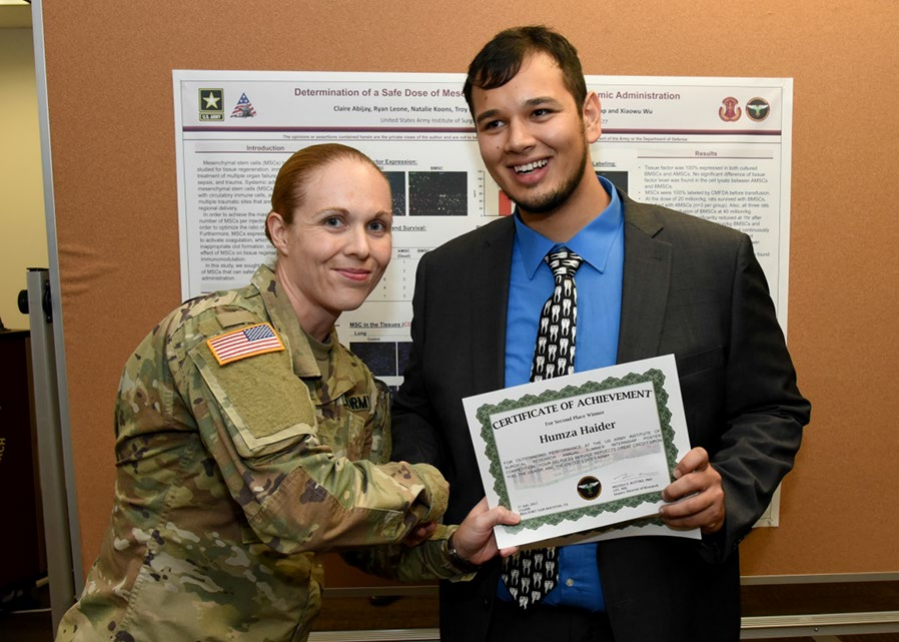
Summer intern presenting work to (left)Capt. Melissa Kottke (Photo credit: US Army Photo by Dr. Steven Galvan, US Army Institute of Surgical Research Public Affairs)

**Application website**

http://usaisr.amedd.army.mil/2016_undergrad_internship.html (accessible from the USA). Here is an alternative source of information about the internship, which can be accessed outside of the USA: https://orise.orau.gov/about/media-center/news-features/2016/army-institute-surgical-research-summer-internships-accepting-applications.html.

## P1 Simultaneous measurement of thrombin and plasmin activity: the thrombin / plasmin ratio

### Ryan Leone^1^, Claire Abijay^2^, Troy Dolmetsch^3^, Natalie Koons^4^, Daniel N. Darlington^5^, Andrew Cap^5^, Xiaowu Wu^5^

#### ^1^Univeristy of Pennsylvania, Philadelphia, PA 19104, USA; ^2^Georgetown University, Washington, DC 20057, USA; ^3^East Tennessee State University, Johnson City, TN 37614, USA; ^4^Mercyhurst University, Erie, PA 16504, USA; ^5^US Army Institute of Surgical Research, JBSA Fort Sam Houston, TX 78234, USA

##### **Correspondence:** Xiaowu Wu (Xiaowu.wu.civ@mail.mil)

*Journal of Translational Medicine* 2017, **15(Supp 4)**:P1

**Background**

Polytrauma and hemorrhage can lead to acute traumatic coagulopathy (ATC), a state of reduced coagulation characterized by increased prothrombin time and internalized normalized ratio [INR(what is INR?)] in patients [1]. Coagulopathic patients have a higher mortality rate than patients with similar injuries and no coagulopathy [2]. The cause of ATC is not well defined, but may be associated with imbalanced blood levels of thrombin and plasmin. Therefore, the ratio of thrombin and plasmin activity may be a valuable diagnostic parameter for ATC. Traditionally, separate colorimetric or fluorogenic substrates are used to measure thrombin and plasmin activity. In this study, we tested substrates that are acted upon by both thrombin and plasmin. An assay was developed to simultaneously simultaneously measure both activities the activities and ratios of thrombin and plasmin;

**Materials and methods**

Multiple concentrations of human thrombin and plasmin were used in this study. SN-20 (fluorogenic substrate, 50 µM) and S-2366 (colorimetric substrate, 2 mM) were used to measure both thrombin and plasmin activity respectively. The fluorescence intensity and absorbance were measured at 350/470 (excitation/emission) for SN-20 and 405 nm for S-2366 using a spectrophotometer with kinetic measurement for 30 min or 1 h. Human recombinant hirudin (25 µg/ml) and aprotinin (10 nM) were used to inhibit thrombin and plasmin respectively. The reaction volume was 100; 20 µl of samples (thrombin, plasmin or plasma), 5 µl inhibitors, 25 µl H_2_O buffer, and 50 µl substrate. Thrombin, plasmin and inhibitors were incubated at room temperature for 10 min before adding the substrates. S-2366 was also tested in human plasma collected from three different donors.

**Results**

Both thrombin and plasmin activity can be measured with SN-20 and S-2366. The combination of thrombin and plasmin had an additive response in pure assay systems with both substrates. Hirudin and aprotinin significantly inhibited thrombin and plasmin activity, respectively. However, hirudin potentiated plasmin activity and aprotinin potentiated thrombin activity, which could interfere with the accuracy of measured thrombin/plasmin ratios. When thrombin and plasmin activity were measured in human plasma, the additive response was shown as well. Hirudin didn’t significantly potentiate plasmin in human plasma.

**Conclusions**

This study suggests that using a common substrate for both thrombin and plasmin can measure their activity simultaneously. This may help develop an assay that can both measure activity and create a ratio of thrombin/plasmin, which can be an important diagnostic tool for ATC. Future studies will focus on optimizing the inhibitor dose and verifying this assay in animal models and human trauma subjects.

**References**

1. Kushimoto S, Kudo D, Kawazoe Y. Acute traumatic coagulopathy and trauma-induced coagulopathy: an overview. J Intensive Care. 2017;5(6).
2. Niles S, McLaughlin D, Perkins J, Wade C, Spinella P, Holcomb J. Increased mortality associated with the early coagulopathy of trauma in combat casualties. J Trauma. 2008;64(6):1459–63.

## P2 PGI_2_ modulates cAMP levels during aggregation in platelets

### Natalie Koons^1^, Ryan Leone^2^, Claire Abijay^3^, Troy Dolmetsch^4^, Xiaowu Wu^5^, Andrew P. Cap^5^, Daniel N. Darlington^5^

#### ^1^Mercyhurst University, Erie, PA 16504, USA; ^2^Univeristy of Pennsylvania, Philadelphia, PA 19104, USA; ^3^Georgetown University, Washington, DC 20057, USA; ^4^East Tennessee State University, Johnson City, TN 37614, USA; ^5^US Army Institute of Surgical Research, JBSA Fort Sam Houston, TX 78234, USA

##### **Correspondence:** Daniel N. Darlington (Daniel.n.darlington.civ@mail.mil)

*Journal of Translational Medicine* 2017, **15(Supp 4)**:P2

**Background**

Trauma and hemorrhage leads to coagulopathy associated with a decrease in platelet function [1]. Because platelets make up 70–80% of clot strength, evaluating the intracellular mechanism controlling aggregation in platelets may lead to a resuscitative strategy that mitigates the coagulopathy. Platelet aggregation is regulated by various intracellular intermediates, including cAMP and cGMP. These two mediators have strong inhibitory effects on platelet aggregation [2]. PGI_2_ is a stimulator of cAMP that is released from endothelial cells during stress [3]. Platelets moving into areas of stressed endothelial cells (trauma) would be affected by the high concentrations of PGI_2_. The objective of this study was to determine the interaction between PGI_2_ and normal agonists of platelet aggregation (ADP, collagen, thrombin [TRAP], and thromboxane [U46619]).

**Materials and methods**

Blood samples were obtained from normal healthy adult volunteers in accordance with institutional standard operating procedures. The blood samples were centrifuged at 200*g* for 10 min, without brakes, to generate platelet rich plasma (PRP). . Samples were then treated with 10 μM PGI2 (prostaglandin I2), or 1 mM of ADP, TRAP, U46619, or collagen (1 mg/ml) for 5 min. Cyclic AMP and cGMP were extracted from 100 μl of PRP after addition of 1 ml EtOH and 10 mM ammonium formate..10μ g/ml cGMP-Br was included as an internal control. Samples were vortexed and centrifuged. The supernatant dried and brought up in 200 μl of 0.1% formic acid. The samples were then analyzed by mass spectroscopy in the tandem mode generating 4 daughters per analyte.

**Results**

Human platelets illustrated a significant elevation in cAMP, but not cGMP, after 5 min incubation with PGI2. ADP, collagen, TRAP and U46619 had no effect on either cAMP or cGMP. TRAP and U46619 attenuated the change in cAMP stimulated by PGI2. ADP and Collagen had little effect on cAMP stimulated by PGI2. None of these agonists had any effect on cGMP.

**Conclusions**

Platelet aggregation can be inhibited by elevations in cAMP. PGI_2_ released from stressed endothelial cells has the potential to modify the ability of platelets to aggregate in response to normal stimuli. These data demonstrate that collagen, thrombin, thromboxane and ADP modulate the PGI_2_ induced elevation of cAMP. More importantly, the presence of PGI_2_ will elevate cAMP and counteract the abilities of ADP, collagen, thrombin or thromboxane toto cause aggregation.

**References**

1. Darlington D, Craig T, Gonzales M, Schwacha M, Cap A, Dubick M. aCOT. Shock. 2013;39(5):440–6.
2. Smolenski A. Novel roles for cAMP/cGMP-dependent signaling in platelets. J Throm Haemostasis. 2015;10: 167–76.
3. Rivera J, Mlozano M, Navarro-Nunez L, Vicente V. Platelet receptors and signaling in the dynamics of thrombus formation. Haematologica. 2009;94: 700–11.

## P3 Determination of a safe dose of mesenchymal stem cells for systemic administration

### Claire Abijay^1^, Ryan Leone^2^, Natalie Koons^3^, Troy Dolmetsch^4^, Daniel N. Darlington^5^, Andrew P. Cap^5^, Xiaowu Wu^5^

#### ^1^Georgetown University, Washington, DC 20057, USA; ^2^Univeristy of Pennsylvania, Philadelphia, PA 19104, USA; ^3^Mercyhurst University, Erie, PA 16504, USA; ^4^East Tennessee State University, Johnson City, TN 37614, USA; ^5^US Army Institute of Surgical Research, JBSA Fort Sam Houston, TX 78234, USA

##### **Correspondence:** Xiaowu Wu (Xiaowu.wu.civ@mail.mil)

*Journal of Translational Medicine* 2017, **15(Supp 4)**:P3

**Background**

Mesenchymal stem cells (MSCs) have been widely studied for tissue regeneration, immunomodulation, and treatment of multiple organ failure. Systemic administration of mesenchymal stem cells (MSCs) allows MSCs to be delivered to multiple traumatic sites that cannot be reached through regional delivery. However, MSCs express tissue factor, which is known to activate hemostasis [1]. This pro-coagulant capability of MSCs may lead to a safety concern by potentially causing inappropriate clot formation and counteracting the beneficial effects of MSCs on tissue regeneration and immunomodulation.

**Materials and methods**

Bone marrow-derived (BMSC) and adipose-derived (AMSC) MSCs were isolated from femur and tibia, and visceral fat of Sprague Dawley rats (300–350 g). MSCs were passaged 2–5 times and labeled with green fluorescent chloromethyl derivatives of fluorescein diacetate. Tissue factor expression was measured by immunohistochemistry and by ELISA. Under anesthesia, rats were cannulated, and MSCs were transfused through the femoral vein at doses of 2.5, 5, 10, 20, and 40 million cells/kg. Citrated whole blood was collected before, immediately after infusion, 1, and 3 h after infusion. The lung, heart, liver, kidney, spleen, and skeletal muscle were taken immediately after euthanasia and stained for platelets (CD61) and nuclei (DAPI).

**Results**

At the dose of 20 million cells/kg, rats survived after infusion with BMSCs; however rats infused with AMSCs did not survive (n = 3 per group). All three rats survived with infusion of BMSCs at 40 million cells/kg. Platelet counts were significantly reduced at 1 h after MSC infusion at doses of ≥ 10 million cells/kg BMSCs and ≥ 5 million cells/kg AMSCs. At doses of 5 and 10 million cells /kg, transfusion of AMSCs led to lower platelet counts as compared to BMSCs. Both AMSCs and BMSCs were found in the lung after infusion. Platelets aggregates were also found in the lung, and varied proportionally with increasing dosages of MSC. Platelet aggregates were found in the right ventricle of the heart after infusion of 40 million cells/kg BMSCs and 20 million cells/kg AMSCs. Few MSCs were found in spleen, liver, kidney and skeletal muscle at any doses of MSCs. Less platelets were found in the spleens after MSC infusion as compared to control.

**Conclusions**

This study suggests that BMSCs have less impact than AMSCs on hemostasis. BMSCs at the dose of 5 million/kg or less have the least risk of causing thrombocytopenia, platelet aggregation and infiltration in lung. The potential risk of systemic administration of MSCs, as characterized by increased thrombosis in the tissue, may be due to tissue factor expression of MSCs.

**Reference**

1. Tatsumi K, Ohashi K, Matsubara Y, Kohori A, Ohno T, Kakidachi H, Horii A, Kanegae K, Utoh R, Iwata T, Okano T. Tissue factor triggers procoagulation in transplanted mesenchymal stem cells leading to thromboembolism. Biochem Biophys Res Commun. 2013;431: 203–9.

## P4 Severe trauma leads to a fall in triphosphates and rise in cAMP in platelets

### Troy Dolmetsch^1^, Natalie Koons^2^, Ryan Leone^3^, Claire Abijay^4^, Andrew P. Cap^5^, Xiaowu Wu^5^, Daniel N. Darlington^5^

#### ^1^East Tennessee State University, Johnson City, TN 37614, USA; ^2^Mercyhurst University, Erie, PA 16504, USA; ^3^Univeristy of Pennsylvania, Philadelphia, PA 19104, USA; ^4^Georgetown University, Washington, DC 20057, USA; ^5^US Army Institute of Surgical Research, JBSA Fort Sam Houston, TX 78234, USA

##### **Correspondence:** Xiaowu Wu (Xiaowu.wu.civ@mail.mil) and Daniel N. Darlington (Daniel.n.darlington.civ@mail.mil)

*Journal of Translational Medicine* 2017, **15(Supp 4)**:P4

**Background**

Severe trauma and hemorrhage lead to a coagulopathic condition characterized by an increase in prothrombin time, and a decrease in platelet function and clotting firmness [1,2]. As platelets constitute 70–80% of clot strength, evaluating the intracellular mechanism controlling aggregation in platelets may lead to a resuscitative strategy that mitigates the coagulopathy. The fall in platelet function could be due to a drop in energy function (ATP) or a rise in inhibitors, including cyclic adenosine monophosphate (cAMP) and cyclic guanosine monophosphate (cGMP).

**Materials and methods**

Polytrauma was induced in isoflurane anesthetized Sprague-Dawley rats (n = 10 per group) by damaging the small intestines, right and medial lobes of the liver, the right leg skeletal muscle, by fracturing the right femur, and then performing 40% hemorrhage. Blood samples were taken before, immediately after, and at 2 and 4 h after trauma. Platelet rich plasma (PRP) was obtained by centrifugation of whole blood at 150 G for 10 min, no brakes. Cyclic AMP and cGMP were extracted from 100 μl of PRP after addition of 1 ml of ethanol, 10 mM ammonium formate, with 10 μg/ml cGMP-Br as an internal control. Triphosphates were extracted from another 100 μl PRP sample by addition of 50% ethanol, 500 mM formic acid. Samples were centrifuged at 20,000 *g* for 10 min, and the supernatant was then dried. All samples were brought up in 200 μl of 0.1% formic acid for analysis by Tandem Mass Spectroscopy. Data were analyzed by One-Way ANOVA corrected for repeated measures N = 10/group. * = P < 0.05 compared to controls.

**Results**

We found that severe trauma and hemorrhage led to a decrease in intracellular triphosphates (ATP, GTP, and inositol trisphosphate [IP3]). We also found that cGMP fell after trauma/hemorrhage. In contrast, trauma/hemorrhage led to a rise in cAMP.

**Conclusions**

Severe trauma leads to a coagulopathy that is associated with a dysfunction in platelet aggregation. We have found that the dysfunction in platelet aggregation could be related to a (1) fall in high energy phosphates (ATP, GTP) as platelets require chemical energy for all aspects of aggregation, (2) a fall in IP3 as this intracellular intermediate acts to release Ca^2+^ to initiate aggregation, and (3) an elevation in cAMP which is known as a direct inhibitor of aggregation.

**References**

1. Darlington D, Craig T, Gonzales M, Schwacha M, Cap A, Dubick M. aCOT. Shock. 2013;39(5): 440–6.
2. Wu X, Darlington D, Cap A. Procoagulant and fibrinolytic activity after polytrauma in rat. Am J Physiol Regul Integr Comp Physiol. 2016;310: R323–9.

## P5 Evaluation of an antimitogenic treatment on the immunomodulatory activity of human mesenchymal stem cells

### Katie J. Jensen^1^, Christopher P. Delavan^2^, Maryanne C. Herzig^2^, Barbara A. Christy^2^, Andrew P. Cap^2^

#### ^1^Rice University, Houston, Houston, TX 77005, USA; ^2^US Army Institute of Surgical Research, JBSA Fort Sam Houston, TX 78234, USA

##### **Correspondence:** Maryanne C. Herzig (maryanne.c.herzig.ctr@mail.mil) and Andrew P. Cap

*Journal of Translational Medicine* 2017, **15(Supp 4)**:P5

**Background**

Mesenchymal stem cells (MSCs) are multipotent cells capable of immunomodulatory activity. To quantify their immunosuppressive capabilities, MSCs are often co-cultured with stimulated peripheral blood mononuclear cells (PBMCs) in a mixed lymphocyte reaction (MLR). Decreased PBMC proliferation, in the presence of MSCs, is considered to be a measure of immunosuppression. However, there are considerable variations in the experimental design of a basic MLR. Strategies to detect PBMC proliferation range from flow cytometry analysis of labeled cell populations to ELISA detection of incorporated bromodeoxyuridine. In these assays, MSCs are often subjected to pre-treatment to arrest cell proliferation before co-culture with PBMCs. To validate the use of a streamlined MLR using a luminescent ATP assay to detect proliferating cells, non-treated MSCs were compared to MSCs mitotically inactivated with Mitomycin C (MMC). The objective of this study was to determine if MSCs treated with an antimitogenic drug are equally capable of suppressing PBMC proliferation as untreated MSCs during co-culture in the presence of phytohemagglutinin A (PHA).

**Materials and methods**

Bone marrow MSCs (BM-MSC) were obtained from commercial sources. PBMCs were isolated from human whole blood and then stored at -80°C until use. MSCs were plated in 96 well plates at defined densities and allowed to attach overnight in MSC media. MSCs treated for 3 h with MMC were then washed with dulbecco’s phosphate buffered saline. MSCs were switched to MLR media (RPMI with 10% FBS, 10 mM Hepes). PBMCs were added at 150,000 per well and PBMC proliferation was stimulated by addition of PHA to a final concentration of 5 µg/ml. Controls included MSCs without PBMCs and/or PHA and PBMCs without MSCs and/or PHA. Proliferation of the PBMCs was determined by analysis of PBMCs in the media; MSC proliferation was measured on cells remaining on the plate after washing. Proliferation correlated with ATP levels and ATP content was measured by the luminescent Cell Titer Glo 2.0 assay (Promega).

**Results**

Preliminary assays of MMC in the 0–20 µg/ml range determined that 10 µg/ml resulted in effective inhibition of MSC proliferation. Using this concentration of MMC for two BM-MSC reactions analyzed in triplicate, proliferation of PBMCs stimulated by PHA was inhibited by both mitotically active and inactive MSCs. However, non-MMC treated MSCs showed higher levels of immunosuppression.

**Conclusions**

Mitomycin C inhibited proliferation of MSCs; however, MMC-treated MSCs were still capable of inhibiting PBMC proliferation. Immunosuppression by MMC-treated MSCs was decreased in comparison to non-treated MSCs but followed a similar trend, justifying the use of mitotically active cells in the MLR.

**References**

1. Salem B, et al. Quantitative activation suppression assay to evaluate human bone marrow-derived mesenchymal stromal cell potency. Cytotherapy. 2015;17: 1675–86.
2. Ryan J, et al. Interferon-ɣ does not break, but promotes the immunosuppressive capacity of adult human mesenchymal stem cells. Clin Exp Immunol. 2007;149: 353–63.

## P6 Static dimensional comparison of low-fidelity airway trainers to Human Morphometrics

### Kelley M. Kempski^1,2^, August N. Blackburn^3^, Robert A. De Lorenzo^1,4^, Megan B. Blackburn^1^

#### ^1^Tactical Combat Casualty Care Research Task Area, US Army Institute of Surgical Research, JBSA Fort Sam Houston, TX 78234, USA; ^2^Department of Biomedical Engineering, University of Delaware, Newark, DE 19716, USA; ^3^South Texas Diabetes and Obesity Institute, University of Rio Grande Valley, Brownsville, TX 78520, USA; ^4^University of Texas Health Science Center at San Antonio, TX 78229, USA

##### **Correspondence:** Megan B. Blackburn (megan.b.blackbrun2.civ@mail.mil)

*Journal of Translational Medicine* 2017, **15(Supp 4)**:P6

**Background**

Appropriate airway management is critically important in trauma patients. Airway compromise is the second leading cause of potentially survivable death on the battlefield, accounting for 1 in 10 preventable deaths [1,2]. Airway management trainer manikins are frequently used in research and for training purposes, and are often the only form of airway training for combat medics in the US military. However, only one study has previously assessed the anatomic accuracy of training manikins and patient simulators [3]. The goal of this study was to quantify the anatomic accuracy of low-fidelity airway trainer manikins used by the US military.

**Materials and methods**

CT scans were taken of SynDaver (Standard Adult Airway Trainer), Laerdal (Airway Management Trainer), and AirSim (AirSim Advance Model) manikins. Manikins were then cut down the sagittal midline using a bandsaw. CT and hand (ruler and goniometer) measurements were compared to human airway measurements obtained from the literature. Manikin measurements were taken three times and averaged to optimize measurement accuracy. The manikin measurements were then scored as a percentile among the same measurements in the human population by assuming the human measurements were normally distributed. More than 50 measurements were taken either by hand or CT scan.

**Results**

On average, manikin measurements fell well outside the 50th percentile relative to the previously published human morphometric data. A small sampling of the measurements taken is shown in Table 1. The airway volumes (nasopharynx, oral pharynx, and total volume) were too large in all three manikins, the oral pharyngeal distances were too long (e.g., epiglottis tip to the posterior pharyngeal wall) in the SynDaver and Leardal, and the SynDaver and Laerdal had a wide right main stem bronchus angle.

**Table 1:** Measurements for the Syndaver, Laerdal, and SimAir manikins obtained from hand measuring* and CT scans compared to human measurements from literature

	Human	SynDaver*	SynDaver CT	Laerdal*	Laerdal CT	SimAir*	SimAir CT
Height mouth opens (cm)	4.78±0.83	5.98 (0.93)	–	4.52 (0.38)	–	8.10 (0.99)	–
Neck circumference (cm)	36.6±3.5	33.4 (0.18)	36.02 (0.43)	43.55 (0.98)	42.84 (0.96)	41.23 (0.91)	40.15 (0.85)
Tracheal length (cm)	8.6±1.1	9.54 (0.8)	9.04 (0.66)	3.27 (0.0)	3.30 (0.0)	7.83 (0.24)	8.01 (0.30)
Epiglottis tip—posterior pharyngeal wall (cm)	0.9±0.4	1.67 (0.97)	1.54 (0.95)	3.54 (0.99)	2.35 (0.99)	1.48 (0.93)	1.10 (0.69)
Angle right main stem bronchus (°)	25	49.57	53.80	29.15	32.37	19.67	–
Total airway volume (cm^3^)	22.5±9.0	–	106.72 (0.99)	–	170.18 (0.99)	–	104.84 (0.99)
Oral pharynx volume (cm^3^)	3.6±2.6	–	20.06 (0.99)	–	27.37 (0.99)	–	7.99 (0.95)

Values are mean (accuracy to human measurement)

**Conclusions**

The SynDaver, Laerdal, and SimAir manikins do not have anatomically correct static dimensions. These inaccuracies may lead to imprecise airway device development, negatively affect training, and cause over-confidence in providers. Future studies will compare manikin hand and CT measurements to a database of human CT scans and quantify the accuracy of manikin’s material properties in order to create a more anatomically accurate airway trainer manikin to be used by the US military.

**References**

1. Bellamy RF. The causes of death in conventional land warfare: implications for combat casualty care research. Mil Med. 1984;149(2):55–62.
2. Eastridge BJ, Mabry RL, Seguin P, Cantrell J, Tops T, Uribe P, et al. Death on the battlefield (2001–2011): implications for the future of combat casualty care. J Trauma Acute Care Surg. 2012;73(6 Suppl 5):S431–7.
3. Schebesta K, Hüpfl M, Rössler B, Ringl H, Müller MP, Kimberger O. Degrees of reality. Anesthesiology. 2012;116(6):1204–9.

## P7 Developing western techniques using WES ProteinSimple: analyzing BDNF and β-actin in rat brain

### Matthew C. Donald^1^, Harold G. Klemcke^2^

#### ^1^Mississippi State University, Mississippi State, MS 39762, USA; ^2^Tactical Combat Casualty Care Research Task Area, US Army Institute of Surgical Research, JBSA Fort Sam Houston, TX 78234, USA

##### **Correspondence:** Harold G Klemcke (Harold.g.klemcke.ctr@mail.mil)

*Journal of Translational Medicine* 2017, **15(Supp 4)**:P7

**Background**

Brain Derived Neurotrophic factor (BDNF) regulates respiration via effects on respiratory frequency, tidal volume and minute volume [1, 2]. BDNF also regulates cardiovascular function via signaling in autonomic nuclei of the brainstem (pons and medulla) [3, 4]. Moreover, ketamine is known to stimulate release of BDNF [5]. In order to examine potential involvement of BDNF in response to ketamine treatment expression levels of BDNF in the rat pons and medulla were quantified [6]. The objective of this study was to optimize the assay conditions for antibody and protein concentration, and incubation times. These results will be used in future studies of the effects of ketamine treatment on expression of BDNF in the brain.

**Materials and methods**

Previously obtained rat brain tissue was homogenized in RIPA buffer containing protease inhibitors. After homogenization tissue lysates were centrifuged at 14,000×*g*_av_. Protein concentrations were determined in supernatants using the Pierce BCA protein assay. Supernatants were saved for Western blot analysis using ProteinSimple WES.

**Results**

β-actin was evaluated as a housekeeping protein for normalization of BDNF expression. β-actin protein expression was evident at an apparent molecular size of 48-kDa. β-actin concentrations increased linearly at antibody (AB; Novus Biologicals, Littleton, CO) concentrations of 1:100 and 1:200. BDNF expression was tested at a single AB (EMD Millipore Corp, Temecula, CA) concentration of 1:50, with low expression (33-kDa) being detected for multiple protein concentrations (0.33, 0.66, and 1.32 μg;). Because of suspected difficulties with extracting BDNF from tissue, a new buffer system and tissue extraction procedures were tested. These involved use of guanidine HCl, tissue sonication, and ultra-centrifugation at 45,000×*g*_av_. Using these procedures, peaks were evident at 11–12, 29, and 35 kDa (Fig. 1).

**Fig. 1:**
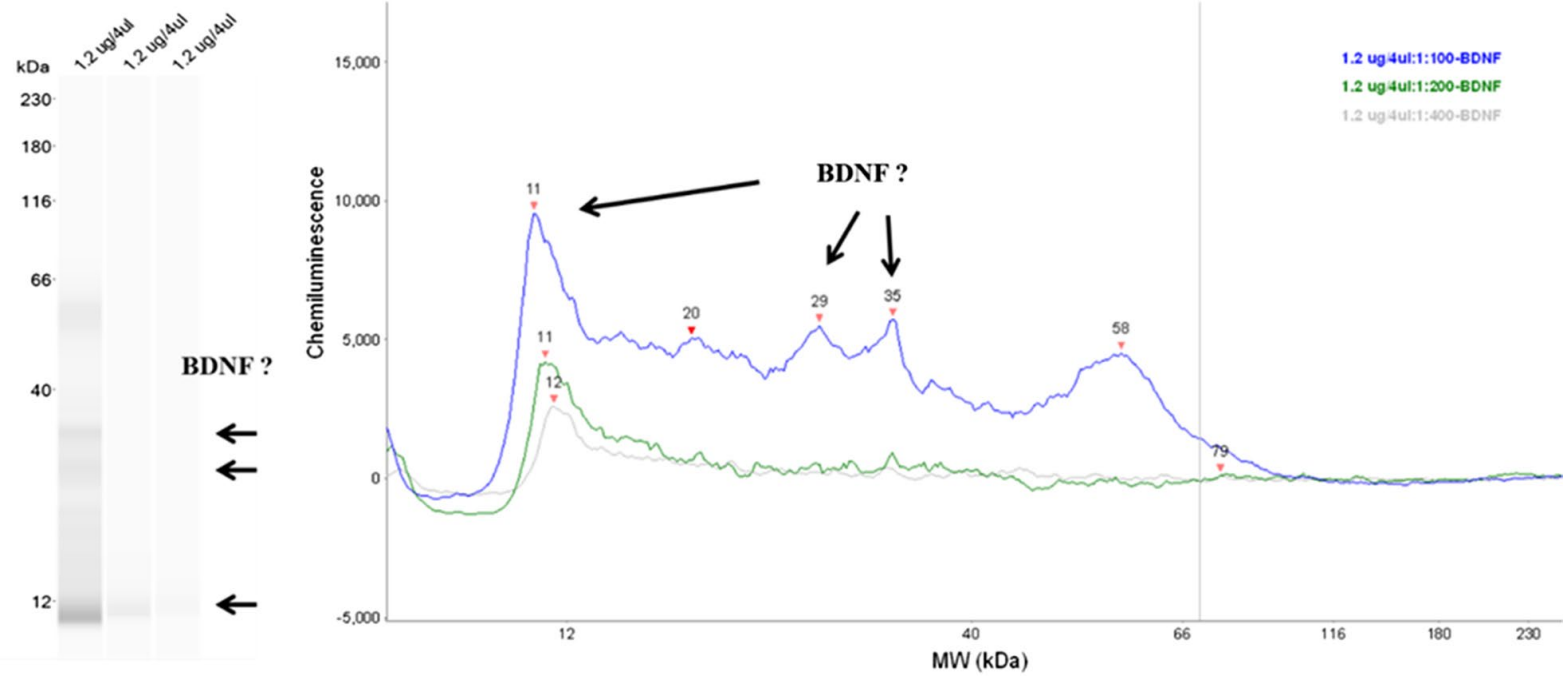
BDNF expression using guanidine HCl extraction procedures. BDNF AB at 1:100, 1:200, and 1:400. Total protein 1.2 μg/4 μl

**Conclusions**

The ProteinSimple WES has improved ease of use and protein sensitivity compared to a traditional western blot. β-actin in our results appeared at 48 kDa, which is consistent with reported WES results. BDNF has proven to be a problematic protein to isolate and quantify. Research is ongoing to improve accuracy of results.

**References**

1. Erickson JT, Conocer JC, Borday V, Champagnat J, Barbacid M, Yancopoulos G, Katz DM. Mice lacking brain-derived neurotrophic factor exhibit visceral sensory neuron losses distinct from mice lacking NT4 and display a severe developmental deficit in control of breathing. J Neurosci. 1996;16(17): 5361–71.
2. Caravagna C, Soliz J, Seaborn T. Brain-derived neurotrophic factor interacts with astrocytes and neurons to control respiration. Eur J Neurosci. 2013;38(9): 3261–9.
3. Clark CG, Hasser EM, Kunze DL, Katz DM, Kline DD. Endogenous brain-derived neurotrophic factor in the nucleus tractus solitarius tonically regulates synaptic and autonomic function. J Neurosci. 2011;31(34): 12318–29.
4. Rothman SM, Griffioen KJ, Wan R, Mattson MP. Brain-derived neurotrophic factor as a regulator of systemic and brain energy metabolism and cardiovascular health. Ann NY Acad Sci. 2012;1264: 49–63.
5. Lepack AE, Fuchikami M, Dwyer JM, Banasr M, Duman RS. BDNF release is required for the behavioral actions of ketamine. Int J Neuropsychopharmacol. 2014;18 (1): 1–6.
6. Pilowsky PM, Lung MSY, Spirovski D, McMullan S. Differential regulation of the central neural cardiorespiratory system by metabotropic neurotransmitters. Phil Trans R Soc Lond B. 2009;364(1529): 2537–52.

## P8 Enteral fluid resuscitation alters splenic function and leukocyte populations post-burn

### Brenna K. Harrington^1^, Belinda I. Gómez^1^, Tony Chao^1^, Joshua S. Little^1^, Tiffany C. Heard^1^, Michael A. Dubick^1^, David M. Burmeister^1^

#### ^1^Damage Control Resuscitation Task Area, US Army Institute of Surgical Research, JBSA Fort Sam Houston, TX 78234, USA

##### **Correspondence:** David M. Burmeister (david.m.burmeister3.civ@mail.mil)

*Journal of Translational Medicine* 2017, **15(Supp 4)**:P8

**Background**

Extensive burn injury (> 30% total body surface area [TBSA]) leads to significant organ damage associated with inflammation. The spleen is a major reservoir of lymphocytes, which are suppressed post-burn, leading to barriers in treatment and recovery [1]. Fluid resuscitation is a critical component of burn treatment and may be utilized to improve splenic recovery. While intravenous fluid resuscitation is the current standard for burn care, enteral resuscitation has been shown to be advantageous in improving treatment outcomes [2]. However, the effect of fluid resuscitation on the role of splenic T-cells after burn injury is largely unstudied. The purpose of this study is to investigate the effect of enteral fluid resuscitation on the phenotype of splenic T cells post-burn.

**Materials and methods**

Twenty-four anesthetized Yorkshire swine were subjected to 40% TBSA thermal burns by 100 °C brass probes [3]. Animals were randomized to four groups; no enteral fluids, ad lib water, volume-matched Oral Rehydration Salt (ORS) from the World Health Organization, and limited-volume (15 ml/kg/day) ORS (n = 6/group). Computerized tomography (CT) scans were performed before and 48 h post-burn, at which point spleens were harvested. Immunohistochemistry was performed to localize and quantify the levels of CD3 and CD4, while hematoxylin and eosin staining was performed to measure the proportion of red and white pulp. Western blots were performed to quantify CD3 protein levels in the spleen. Quantification of the inflammatory cytokines TLR4 and IL-8, were determined through qPCR. Significance was determined when p < 0.05.

**Results**

CT reconstructions indicate little to no change in the splenic artery diameter of swine treated with volume-matched ORS (−0.03 ± 0.27 mm), while swine treated with no water exhibit a dramatic reduction in diameter post-burn (−1.7 ± 0.59 mm) over the 48 h period. Increased levels of white pulp were highest in the ad lib water group (233.0 ± 1.7 AU) and volume-matched ORS (229.7 ± 2.5 AU) compared to no fluids (221.9 ± 5.2 AU) or low-volume ORS (218.7 ± 3.4 AU). Ad lib water increased levels of CD3 protein, which was localized surrounding white pulp. Although TLR-4 and IL-8 expression tended to be down-regulated with ORS access, but the differences were not significant.

**Conclusions**

Burn injury leads to significant disruption of leukocytes within the spleen, which can be altered with enteral resuscitation. Specifically, access to ad lib water or ORS increases splenic lymphocytes post-burn, and may be anti-inflammatory. Enteral fluids also preserve splenic perfusion, with minimal change in the splenic artery diameter. The therapeutic efficacy of enteral resuscitation in burn injury warrants further investigation.

**References**

1. Zhao G, Yu YM, Kaneki M, Bonab AA, Tompkins RG, Fischman AJ. Simvastatin reduces burn injury-induced splenic apoptosis via downregulation of the TNF-alpha/NF-kappaB pathway. Ann Surg. 2015;261: 1006–12.
2. Moghazy AM, Adly OA, Elbadawy MA, Hashem RE. Evaluation of who oral rehydration solution (ORS) and salt tablets in resuscitating adult patients with burns covering more than 15% of total body surface area (TBSA). Ann Burns Fire Disasters. 2016;29: 43–7.
3. Burmeister DM, McIntyre MK, Baker BA, Rizzo JA, Brown A, Natesan S, Chung KK, Christy RJ. Impact of Isolated burns on major organs: a large animal model characterized. Shock. 2016;46: 137–47.

## P9 Effect of intravenous fluid resuscitation on cortisol production of the adrenals in burn injury

### Celestine J. He^1,2^, Belinda I. Gómez^1^, Tony Chao^1^, Joshua S. Little^1^, Tiffany C. Heard^1^, Michael A. Dubick^1^, David M. Burmeister^1^

#### ^1^Damage Control Resuscitation Research Task Area, US Army Institute of Surgical Research, JBSA Fort Sam Houston, TX 78234, USA; ^2^Department of Biological Sciences, Northwestern University, Evanston, IL 60201, USA

##### **Correspondence:** David M. Burmeister (david.m.burmeister3.civ@mail.mil)

*Journal of Translational Medicine* 2017, **15(Supp 4)**:P9

**Background**

Cortisol is a steroid hormone involved in the stress response that is synthesized from cholesterol in the adrenal gland. Severe thermal injury to the body induces a state of metabolic and physiological stress [1], prompting the hypothalamic-pituitary-adrenal (HPA) axis to increase production of stress hormones. In patients with severe burns (40–50% total body surface area) the extent of burn injury positively correlates to plasma cortisol levels [2], but little is known about adrenal function after burn injury. The objective of this study was to evaluate the effect of intravenous (IV) volume Lactated Ringers on circulating cortisol levels and steroidogenic enzymes within the adrenal gland.

**Materials and methods**

Anesthetized Yorkshire swine sustained 40% TBSA burns from brass probes heated to 100 °C and placed on the skin for 30 s. Burned swine received intravenous (IV) fluid resuscitation with Lactated Ringers (LR) solution at two different volumes: 15 ml × body weight (BW)/day (Limited Volume; LV, n = 3) or 2 ml × 40 (%TBSA) × BW/day (Modified Brooke; MB, n = 3). Non-burned animals (sham; n = 3) were also water restricted to induce some level of stress. ELISA was used to quantify circulating cortisol levels from plasma at baseline, 6, 24, and 48 h following burns. Animals were sacrificed 48 h post-burn and adrenal glands were harvested for quantification of mRNA, Western blot, and histopathology.

**Results**

Gross morphology of adrenal glands was similar amongst treatment groups. Plasma cholesterol levels were highest in the sham swine at 48 h (p < 0.05), followed by LV and MB. Plasma cortisol levels, however, were highest in the MB animals at 48 h (p < 0.05), followed by LV and sham. Gene expression of cleavage enzyme CYP21 was up-regulated only in MB (p = 0.11), whereas expression of additional enzymes along the cortisol synthesis pathway were not regulated. Western blot demonstrated increased concentrations of Caspase-3 (p = 0.009) in MB when compared to LV and sham groups. Histopathology revealed more white blebbing in the medulla of the adrenals in LV and MB treatments than in the sham.

**Conclusions**

This study demonstrates that the adrenal gland integrity is compromised following burn injury and that large volumes of LR IV fluid exacerbate adrenal insufficiency. CYP21 may be the rate limiting enzyme required for cortisol synthesis after traumatic injury. Future work will include a larger sample size and quantifiable measurements of necrosis and volume size of the adrenal gland.

**References**

1. Yang C, et al. Effects of bilateral adrenalectomy on the innate immune responses following trauma in rats., Injury, Int. J. Care Injured. 2011;42: 905–12.
2. Wise L, et al. Adrenal cortical function in severe burns. Arch Surg. 1972;105: 213–20.

## P10 Mesenchymal stem cells in the setting of acute respiratory distress syndrome

### Amy Xu^1,2^, Kerfoot Walker III^1,3^, Arezoo Mohammadipoor^1,3^, Luis Rodriguez^1,3^, Teryn Roberts ^1,4^, Andriy Batchinsky^1,4^, Leopoldo Cancio ^1^, Ben Antebi^1^

#### ^1^United States Army Institute of Surgical Research, JBSA Fort Sam Houston, Houston, TX, USA; ^2^Stanford University, Stanford, CA, USA; ^3^Oak Ridge Institute for Science and Education, Oak Ridge, TN, USA; ^4^The Geneva Foundation, Tacoma, WA, USA

##### **Correspondence:** Ben Antebi (ben.antebi.ctr@mail.mil)

*Journal of Translational Medicine* 2017, **15(Supp 4)**:P10

**Background**

One of the defining characteristics of acute respiratory distress syndrome (ARDS) is an imbalance between pro- and anti-inflammatory cytokines [1, 2]. Mesenchymal stem cells (MSCs) have been proposed to possess therapeutic potential to treat ARDS due to their ability to regulate the immune response; consequently MSCs are currently being evaluated in phase 2 clinical trials [3]. The objective of this study was twofold: (1) characterize cytokine expression in serum from ARDS subjects (2) determine MSC function following “pre-conditioning” with ARDS serum. We hypothesized that the regenerative function of MSCs is enhanced following exposure to ARDS serum.

**Materials and methods**

A porcine model of ARDS was used. ARDs was induced in study animals by smoke inhalation and 40% total body surface area full thickness burn. The animals were divided into three cohorts: Uninjured (no ARDS, n = 4), Injured Untreated (n = 5), and Injured Treated (~ 6 million/kg MSCs, n = 7). Serum was collected at baseline, post-injury, 24, 48, 72 h and analyzed with Milliplex for expression of inflammatory mediators. Porcine bone marrow MSCs were subsequently conditioned in 5% pooled serum from the 3 different cohorts for 48 h ex vivo. The ‘pre-conditioned’ media were then analyzed with Milliplex, while the ‘pre-conditioned’ MSCs were analyzed for viability (trypan blue exclusion); proliferation (Picogreen); metabolism (Vybrant); gene expression (qRT-PCR); clonogenicity (CFU-F); and interactions with mononuclear cells. Statistics were performed using a 2-way ANOVA with multiple comparisons to the Uninjured group and Bonferroni correction. A p < 0.05 was considered statistically significant.

**Results**

The serum from the Injured Treated animals had significantly lower levels of IFN-γ (p < 0.05) and significantly higher levels of IL-1ra (p < 0.01) and IL-6 (p < 0.05) (Fig. 1a). Similarly, upon exposure to 5% serum ex vivo, the MSCs exposed to Injured Treated serum secreted higher levels of the anti-inflammatory mediators IL-1ra (p < 0.0001) and IL-10 (p < 0.01) while dampening the secretion of various pro-inflammatory cytokines (Fig. 1b). Additionally, TLR-4 and VEGF were upregulated in the MSCs exposed to Injured Treated serum. Although MSCs treated with the Injured Untreated serum exhibited upregulation of the stem cell genes SOX-2 and Nanog, their proliferative and clonogenic capacities were significantly reduced (Fig. 1c, d).

**Fig. 1:**
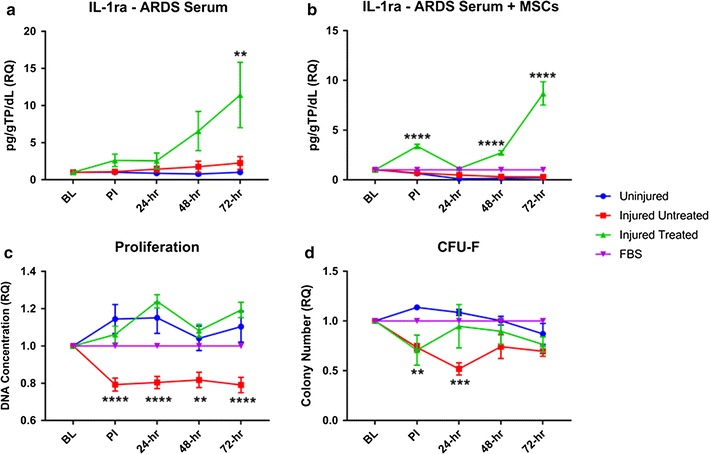
Mesenchymal stem cells (MSCs) function in an acute respiratory distress syndrome (ARDS) milieu. **a** ARDS animals treated with allogeneic MSCs show increased serum levels of interleukin-1 receptor antagonist (IL-1ra); **b** MSCs exposed to ARDS serum from treated animals demonstrate increased levels of IL-1ra; **c** decreased proliferation of MSCs exposed to ARDS serum from untreated animals; **d** decreased clonogenic capacity of MSCs exposed to ARDS serum from untreated animals

**Conclusions**

MSC treatment appears to delay ARDS progression by modulating the inflammatory milieu in vivo. Exposure to ARDS serum ex vivo elucidated the trends seen in vivo, which appear to be mediated, in part, through TLR-4 and VEGF. Other than the altered secretory cytokine profile, serum from subjects with untreated ARDS appears to negatively impact the proliferative function of MSCs.

**References**

1. Matthay MA, Ware LB, Zimmerman GA. The acute respiratory distress syndrome. J Clin Invest. 2012;122(8): 2731–40.
2. Strieter RM, Kunkel SL. Acute lung injury: the role of cytokines in the elicitation of neutrophils. J Investig Med. 1994;42(4): 640–51.
3. Wilson JG, Liu KD, Zhuo H, Caballero L, McMillan M, Fang X, Cosgrove K, Vojnik R, Calfee CS, Lee JW, et al. Mesenchymal stem (stromal) cells for treatment of ARDS: a phase 1 clinical trial. Lancet Respir Med. 2015;3(1): 24–32.

## P11 Effects of S107 RyR-FKB12 stabilizer on platelet function

### Ryan A. Walford^1,2^, Colby S. McIntosh^1^, Grantham C. Peltier^1^, Umang Sharma^1^, Robbie K. Montgomery^1^, Michael A. Meledeo^1^, James A. Bynum^1^, Andrew P. Cap^1^

#### ^1^Blood and Coagulation Task Area, US Army Institute of Surgical Research, JBSA Fort Sam Houston, TX 78234, USA; ^2^Dwight Look College of Engineering, Department of Biomedical Engineering, Texas A&M University, College Station, TX 77840, USA

##### **Correspondence:** Andrew P. Cap (Andrew.p.cap.mil@mail.mil)

*Journal of Translational Medicine* 2017, **15(Supp 4)**:P11

**Background**

The hemostatic potential of cold-stored platelets rapidly deteriorates due to premature platelet aggregation, limiting platelet storage life to 5 days. Free calcium ions (a key factor in the platelet activation cascade) are thought to be responsible for the decreased function of stored platelets. The compound 2,3,4,5-tetrahydro-7-methoxy-4-methyl-1,4-benzothiazepine (S107) has been found to lower intracellular Ca^2+^ levels in muscle cells by stabilizing oxidized Ryanodine Receptors (RyR). Because platelets share the same RyR channels as found in the sarcoplasmic reticulum of muscle cells, it is hypothesized that administering S107 to plasma can delay aggregation and extend the lifespan of stored platelets. An extended storage life would increase the Army’s capacity for clinical platelet storage. This study evaluates S107’s potential to extend the viability of stored platelets beyond the current 5 days limit [1].

**Materials and methods**

Platelets were collected with TRIMA apheresis and diluted with platelet additive solution (PAS) at a 35:65 plasma:PAS ratio by volume. The platelets were distributed into 6 × 15 ml platelet storage bags and were dosed with 0 (Control), 0.1, 1, 25, 50, and 100 μM S107 and were stored at 4 °C (non-agitated). Stored platelets were analyzed on day 1, 5,10, and 15. Clot formation metrics—clot time (R), clot formation time (CFT), maximum clot strength (MA), and fibrinolysis—were measured by thromboelastography on TEG–5000’s using 15 mM Ca2+ and a 1:5000 dilution of tissue factor agonist. Surface receptor expression was measured on a BD FACSCanto-2 flow cytometer by standard methods. Platelet count was determined using Micros 60 CBC analysis. Lactate metabolics, pH, and HCO_3_ were determined by iSTAT Chem-8 blood analysis. Platelet aggregation was analyzed by optical platelet aggregometry using Chrono–Log aggregometetry with TRAP-6, ADP, and Collagen agonists.

**Results**

By day 10, the S107 groups did not statistically differ from the Control in respects to R-time, CFT, MA, Lactate concentration, or Platelet Count. On average, all dosages had slightly fewer platelets (Average of 1029 plts/ml with a SEM of 28 plts/ml) and an overall increase in Lactate—indicating that fewer platelets had survived and were equally as metabolically active as the control group.

**Conclusions**

S107 failed to provide a significant benefit to either platelet function or preservation. S107 may have stabilized the FKBP12-RyR interaction, as it does in muscle tissue, however it failed to delay platelet activation. This study indicates that S107 alone is an inadequate preservative and will not meet the Army’s demand for improved platelet storage.

**Reference**

1. Yingwu M, Xu L, Kramer H, Tomberlin G, Townsend C, Meissner G. Stabilization of the skeletal muscle ryanodine receptor ion channel-FKBP12 complex by the 1,4-benzothiazepine derivative S107. PLoS ONE. 2013;8(1):1–12.

## P12 Metabolic consequences of room-temperature platelet storage

### Ryan A. Walford^1,2^, Colby S. McIntosh^1^, Grantham C. Peltier^1^, Umang Sharma^1^, Robbie K. Montgomery^1^, Michael A. Meledeo^1^, James A. Bynum^1^, Andrew P. Cap^1^

#### ^1^Blood and Coagulation Task Area, US Army Institute of Surgical Research, JBSA Fort Sam Houston, TX 78234, USA; ^2^Dwight Look College of Engineering, Department of Biomedical Engineering, Texas A&M University, College Station, TX 77840, USA

##### **Correspondence:** Andrew P. Cap (Andrew.p.cap.mil@mail.mil)

*Journal of Translational Medicine* 2017, **15(Supp 4)**:P12

**Background**

Current blood banking procedures for storing clinical platelets recommend room temperature storage (RTS) under agitation. This practice limits the platelet’s functional lifespan to 5 days and promotes bacterial growth. Cold-storage (4 °C) however, is not clinically used because platelets are activated in colder temperatures and clear the body faster than RTS platelets. In response to these concerns, platelet additive solutions (PAS) have been developed to mitigate cold-stored aggregation; and further research suggests that even cold-stored platelets are retained within the body long enough to have lifesaving effects. With cold-stored PAS platelets, activation is minimal and bacterial growth is prevented, allowing platelets to preserve their function longer for clinical use [1, 2].

The objective of this study was to quantify the effects of temperature on platelet function in order to compare storage at 4 °C against current storage standards.

**Materials and methods**

Platelets were collected via TRIMA apheresis and diluted with PAS at a 35:65 platelet:PAS volume ratio. The platelets were distributed into 4 × 15 ml storage bags (2 bags per donor) and stored at 4 °C. After 3 days, 1 bag was moved to an agitator at 22.3 °C. 1 bag was moved to the same agitator after 5 days. The remaining bags remained at 4 °C. Measurements were taken at 1, 5, 10, and 15 days. Clot formation metrics—clot time, formation time, and strength—were measured by TEG–5000 thromboelastography using 15 mM Ca^2+^ and a 1:5000 dilution of tissue factor agonist. Surface receptor expression was measured on a BD FACSCanto-2 flow cytometer. Platelet count was determined by Micros 60 CBC analysis. Lactate metabolics, pH, and HCO_3_ were determined by iSTAT Chem-8 blood analysis. Platelet aggregation was analyzed by optical platelet aggregometry using Chrono–Log aggregometetry with TRAP-6, ADP, and Collagen agonists.  

**Results**

On day 10, the average cold-storage platelet count was 1166 plt/ml as opposed to 1334 for RTS. The difference between average lactate levels (an indicator of metabolic activity) was 6.73 μM (4 °C) and 11.36 μM (RTS). The level of free bicarbonate ions (an indicator of metabolic preservation) was 2.03uM (4 °C), but was too small to calculate for RTS.

**Conclusions**

Although RTS had superior platelet counts, its platelets were far less functional than the fewer numbered cold-stored platelets. In trauma situations, the clearance time of cold-stored platelets is ample to save lives in hemorrhage patients; and can therefore be argued that the preservative and sanitation benefits of cold stored platelets will better meet clinical demands.

**References**

1. Bynum J, Reddoch K, Montgomery R, Peltier G, Taylor A, Chance T, Meledeo M, Ramasubramanian A, Cap A. Mitochondrial health and integrity plays a direct role in the preservation of mitochondrial-associated gene expression and the regulation of apoptosis induction in stored platelets. (n.d.): n. pag. Web. (In process of publication).
2. Reddoch K, Pidcoke H, Montgomery R, Fedyk C, Aden J, Ramasubramanian A, Cap A. Hemostatic function of apheresis platelets stored AT 4-C AND 22-C. United States Army Institute of Surgical Research, JBSA Fort Sam Houston, Houston, TX 23 Oct. 2013. Web.

## P13 Inflammatory signaling and blood cell contact influence tissue factor expression in human mesenchymal stem cells

### Sarah Lovelace^1^, Larry Estlack^3^, Christopher Delavan^3^, Katherine Jensen^2^, Maryanne Herzig^3^, Andrew Cap^3^, Barbara Christy^3^

#### ^1^Trinity University, San Antonio, TX 78212, USA; ^2^Rice University, Houston, TX 77005, USA; ^3^US Army Institute of Surgical Research, JBSA Fort Sam Houston, TX 78234, USA

##### **Correspondence:** Barbara Christy (Barbara.christy3.vol@mail.mil)

*Journal of Translational Medicine* 2017, **15(Supp 4)**:P13

**Background**

Mesenchymal stem cells (MSCs) modulate inflammation and immune function, suggesting that they may reduce secondary damage after injury. Despite potential benefits, our understanding of MSC safety in injured patients is incomplete. MSCs express tissue factor (TF), a key initiator of the blood coagulation cascade. TF levels reflect pro-coagulant activity which could cause thrombosis after intravenous injection of the MSCs [1]. MSCs are known to affect immune cells, but little is known about how immune cells affect MSCs. To understand MSC safety it is important to investigate the effects of inflammatory conditions and blood cell contact. The objectives of this study were to determine if MSC activation by inflammatory signals affects TF expression and if contact with human blood cells could activate MSCs and affect TF expression.

**Materials and methods**

Human bone marrow and adipose MSCswere treated with interferon gamma (IFN-γ) and/or tumor necrosis factor alpha (TNF-α). Levels of secreted indoleamine 2,3-dioxygenase (IDO) product kynurenine in the medium were determined using a colorimetric assay. Expression of TF and IDO genes was evaluated by qRT-PCR, normalized against internal reference gene expression. Cell surface TF expression was measured by flow cytometry. To assess effects of blood cell contact, MSCs were co-cultured with peripheral blood mononuclear cells (PBMCs) with or without mitogenic stimulation. Duplicate wells were harvested for mRNA isolation and flow cytometry.

**Results**

Unlike alveolar epithelial cells [2], MSCs exposed to IFN-γ and TNF-α did not demonstrate a consistent increase in TF expression. Responses varied between different MSCs. Most showed no significant change with treatment. In contrast, IDO dramatically increased after IFN-γ and TNF-α treatment for all MSCs. Kynurenine increased in parallel with IDO mRNA expression. In co-culture experiments, TF mRNA levels in adipose MSCs generally decreased in presence of PBMCs, with and without mitogenic activation. This is evident in both gene and cell surface TF expression. Bone marrow MSCs expressed less TF and were not significantly affected by PBMC presence.

**Conclusions**

Human mesenchymal stem cells respond strongly to treatment with IFN-γ and TNF-α, dramatically upregulating IDO mRNA and enzyme activity. However, TF mRNA is not affected significantly in most MSCs. In contrast, TF mRNA and cell surface TF expression are decreased following co-culture with human PBMCs, especially in adipose MSCs. Because cell surface TF correlates with pro-coagulant activity [1], the decrease seen when allogeneic MSCs come into contact with blood could reduce risk of thrombosis and improve safety. Further investigation is needed to determine the mechanism of action, in order to mimic the effect without the need for co-culture

**References**

1. Christy BA, Herzig MC, Montgomery RK, Delavan C, Bynum JA, Reddoch KM, Cap AP. Pro-coagulant activity of human mesenchymal stem cells. J Trauma Acute Care Surg. 2017;81: S164–9.
2. Bastarache JA, Sebag SC, Grove BS, Ware LB. Interferon-γ and tumor necrosis factor-α act synergistically to up-regulate tissue factor in alveolar epithelial cells. Exp Lung Res. 2011;37: 509–17.

## P14 Characterization of gene expression from different time points in *Pseudomonas aeruginosa*

### Lexi Kazen^1^, Lee C. Mangum^2^, Gerardo R. Garcia^2^, Kevin S. Akers^2,3^

#### ^1^University of Wisconsin, Madison, WI, USA; ^2^US Army Institute of Surgical Research, JBSA Fort Sam Houston, TX, USA; ^3^Brooke Army Medical Center, JBSA Fort Sam Houston, TX, USA

##### **Correspondence:** Kevin S. Akers (kevin.s.akers.mil@mail.mil)

*Journal of Translational Medicine* 2017, **15(Supp 4)**:P14

**Background**

The USAISR clinical isolate collection is the result of nearly 40 years of sample collection and storage. There are approximately 71,000 bacterial isolates currently in storage, the vast majority of which were obtained from patients in the USAISR Burn Unit and are available for clinical research. *P. aeruginosa* is a common infective agent in burn patients and is thus is an important member of the USAISR clinical isolate collection. This collection provides a rare opportunity to perform comparative analysis of virulence factor expression in isolates obtained decades apart, leading to an increased understanding of how these pathogens have evolved over time.

**Materials and methods**

150 *P. aeruginosa* isolates from the clinical specimen library housed within USAISR were randomly selected for viability assessment. A representative sample of 30 viable isolates collected between 1983 and 2003 were chosen for virulence factor expression analysis. Isolates were cultured in media supplemented with 10% human plasma and total RNA was extracted and quantified cDNA was synthesized from sample mRNA. Virulence factor gene expression was analyzed via qRT-PCR.

**Results**

Out of 150 isolates tested for viability, 53 were found to be viable, and a subset of 30 were selected for virulence factor expression analysis with 29 isolates yielding RNA of sufficient quality for qRT-PCR analysis. Significant variation in the expression of serralysin (an alkaline metalloproteinase—aprA), a *P. aeruginosa* virulence response regulator (gacA), and an alginate biosynthetic protein (algX) was observed throughout the 1983 to 2003 period investigated. No clear trends in virulence factor expression over time were observed in the study population.

**Conclusions**

The USAISR clinical isolate collection represents an important resource for the study of clinically relevant pathogens, particularly with regard to changes in pathogen characteristics over time. Virulence factor expression varied greatly over time, suggesting a high degree of genetic heterogeneity among *P. aeruginosa* strains recovered from patients in the USAISR Burn Unit. Despite this difference in expression at various time points, the lack of specific trends over time in regards to gene expression suggests that *P. aeruginosa* infecting burn patients has not undergone a significant phenotypic shift in recent decades. Further retrospective analyses linking virulence factor expression profiles with clinical outcomes over recent years has the potential to inform clinical practice for improved management of burn infections. The evaluation of changes in virulence factor expression profiles within this collection is ongoing.

**Reference**

1. Fujitani, Shigeki, MD, Kathryn S. Moffett, MD, and Victor L. Yu, MD. *Pseudomonas Aeruginosa*. Antimicrobe. E-Sun Technologies; 2010. Web. 7 July 2017.

